# Interference scores have inadequate concurrent and convergent validity: Should we stop using the flanker, Simon, and spatial Stroop tasks?

**DOI:** 10.1186/s41235-020-0207-y

**Published:** 2020-02-13

**Authors:** Kenneth R. Paap, Regina Anders-Jefferson, Brandon Zimiga, Lauren Mason, Roman Mikulinsky

**Affiliations:** Department of Psychology, San Francisco State University, San Francisco, CA 94132 USA

**Keywords:** Inhibitory control, Self-control, Impulsivity, Flanker task, Simon task, Spatial Stroop task, Music, Video gaming, Bilingualism, Sex, Intelligence

## Abstract

**Background:**

Two-hundred one college undergraduates completed four nonverbal interference tasks (Simon, spatial Stroop, vertical Stroop, and flanker) and trait scales of self-control and impulsivity. Regression analyses tested 11 predictors of the composite interference scores derived from three of the four tasks and each task separately. The purpose of the study was to examine the relationships between laboratory measures of self-control, self-report measures, and the degree to which control might be related to extensive experience in activities that logically require self-control.

**Results:**

Fluid intelligence and sex were significant predictors of the composite measure, but bilingualism, music training, video gaming, mindfulness/meditation, self-control, impulsivity, SES, and physical exercise were not.

**Conclusions:**

Common laboratory measures of inhibitory control do not correlate with self-reported measures of self-control or impulsivity and consequently appear to be measuring different constructs. Bilingualism, mindfulness/meditation, playing action video games, and music training or performance provide weak and inconsistent improvements to laboratory measures of interference control. Flanker, Simon, and spatial Stroop effects should not be used or interpreted as measures of domain-general inhibitory control.

## Significance

Individuals and societies are vested in maximizing good choices that enable goal attainment and long-term wellbeing and minimizing impulsive behaviors that yield to temptations and poor choices. Cognitive psychology has developed elaborate models of cognitive control based on performance in exquisitely controlled laboratory tasks. The results of the reviewed published articles in combination with our new results convincingly show that performance in laboratory tasks does not predict self-control in everyday life. These tasks should not be characterized as reflecting “inhibitory control.” Public policy and individual choices are also influenced by claims that certain types of life experience (bilingualism, music performance, playing video games, and mindfulness/meditation) may enhance “inhibitory control.” No compelling evidence has been found for these benefits. Ironically, the weight of the second point is challenged by the first point that these cognitive tasks do not predict cognitive control in everyday life.

## Background

**Fig. 1:**
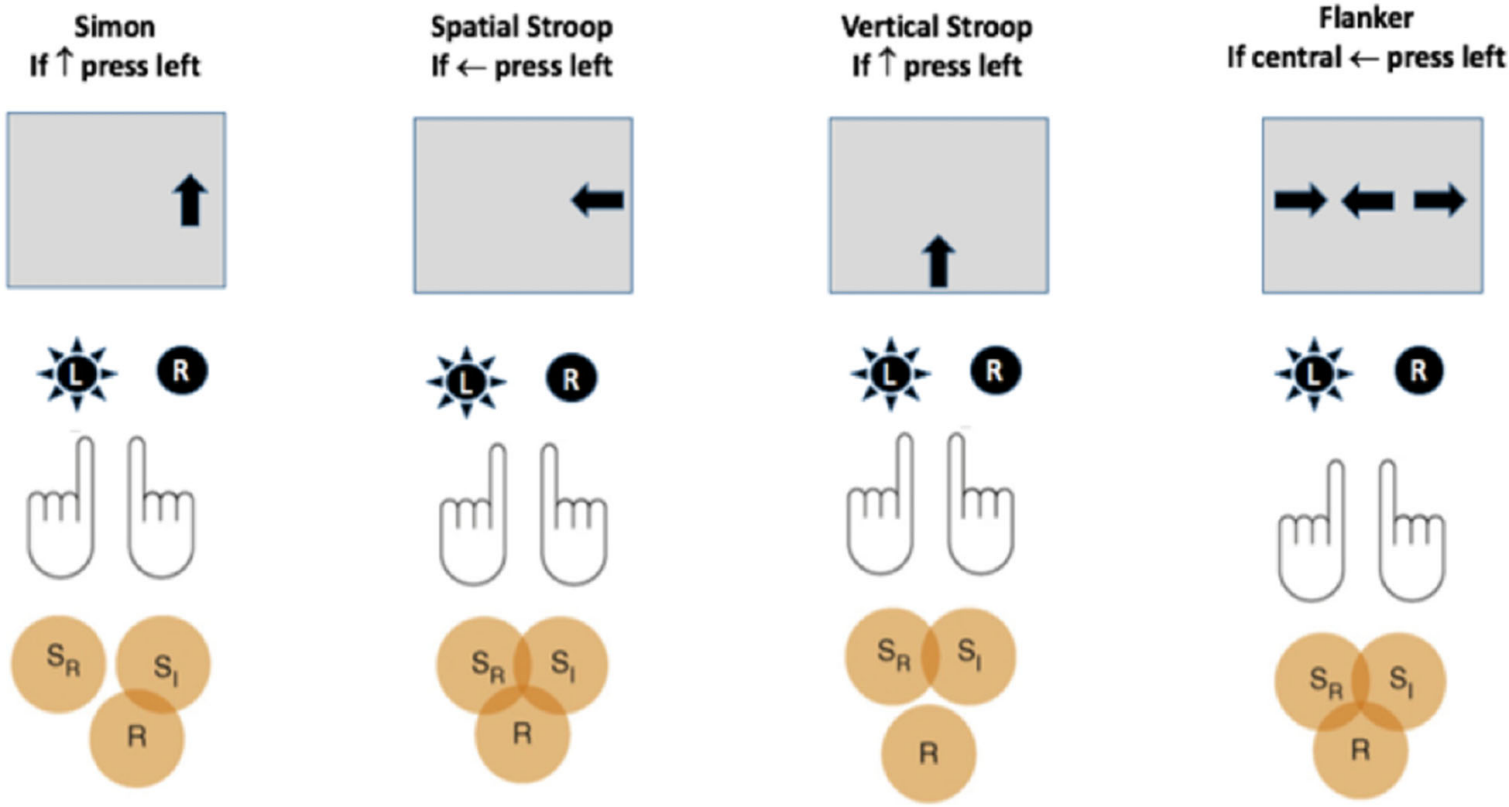
The four nonverbal interference tasks used in the present study. The representational scheme is based on Fig. 1 from Egner (2008). From top to bottom for each task is the name of the task, the response rule, a screen illustrating an incongruent trial, response keys with the correct response radiated, and finally Venn diagrams showing potential conflict between the irrelevant stimulus (S_I_), relevant stimulus (S_R_), and response (R)

Nonverbal interference tasks (like the four illustrated in Fig. 1) have played a leading role in cognitive psychology. The flanker task was introduced by Eriksen and Eriksen (1974), and their article has been cited more than five thousand times. Its hybrid, the attention network test (ANT^1^), was launched in Fan, McCandliss, Sommer, Raz, and Posner (2002) and has been cited nearly three thousand times. The eponymous Simon effect traces back to the Simon (1969) and Simon and Small Jr. (1969) articles that have been cited more than two thousand times. The influential review of the Simon and spatial Stroop task conducted by Lu and Proctor (1994) has nearly a thousand citations. A very conservative search of PsychARTICLES and PsychINFO suggests that more than 4000 articles have not merely cited results from these tasks but have used them.

## What do interference scores reflect?

One major attraction of these tasks is that they appear to isolate and contrast trials where conflict or competition occurs (namely, incongruent trials) from those where conflict is absent (namely, congruent trials). The difference in mean RT between these two trial types should provide a measure of the effectiveness of conflict resolution between groups or individuals. This difference score will be referred to as an *interference score* and is intended to be theoretically neutral. That said, interference scores are often treated as measures of inhibitory control, with smaller scores reflecting better control. But as MacLeod, Dodd, Sheard, Wilson, and Bibi (2003) warned us, this may be confusing a phenomenon (e.g., slower responses on incongruent trials) with a mechanism (e.g., suppressing competing information) because the magnitude of an interference score may be the product of upregulating the task relevant information, inhibiting the irrelevant information, a combination of both, or no control at all. Given this ambiguity, the terms “inhibition” or “inhibitory control” may be misleading with respect to the control mechanism(s) actually recruited during nonverbal interference tasks.

Although inhibitory control (or self-control) is a unitary construct in the common vernacular, cognitive psychologists have long entertained the possibility that it can be fractionated into multiple forms or components. Consider the most influential taxonomy for analyzing task differences: Kornblum’s (1994) Dimensional Overlap Model. The model distinguishes between tasks with stimulus-response (S-R) or stimulus-stimulus (S-S) incompatibility. The incompatibilities of the four tasks used in the present study are illustrated in Fig. 1. For each panel, the S-R rule is at the top, a display representing a correct response on an incongruent trial is in the middle, and the Venn diagrams at the bottom represent, by their intersections, where conflict can be generated and resolved. The first (leftmost) panel is a pure S-R task that is often referred to as a Simon task. A single arrow (pointing either up or down) is presented either to the left or right of fixation, and the rule is to press the left key if the arrow points up and the right key if it points down. Given the natural tendency to react toward the source of stimulation, competition can occur when the physical location and the rule are incongruent, as illustrated by overlap between S_I_ (the irrelevant stimulus) and R (the correct response). Note that no overlap occurs between the task-relevant and task-irrelevant stimulus representations because the arrow’s form varies on an up-down dimension, whereas its location varies on a left-right axis.

If the vertical arrows are displaced either above or below fixation, as in the third panel, the task transforms into a pure S-S task. Because the upward pointing arrow appears below the fixation, the task-relevant dimension (up arrow) is opposite its location (below) and causes S-S incompatibility. No S-R incompatibility occurs because the layout of the response keys (horizontal) is orthogonal to the up-down direction of the arrow. This pure S-S task will be referred to as the vertical Stroop task.

A flanker task is shown at the far right of Fig. 1. In order to reduce the differences between the flanker task and the other three tasks, we included only a single flanker on each side of the central target. When the flankers point in the same direction as the central arrow, the trial is congruent, and when they point in the opposite direction, it is incongruent.

**Fig. 2:**
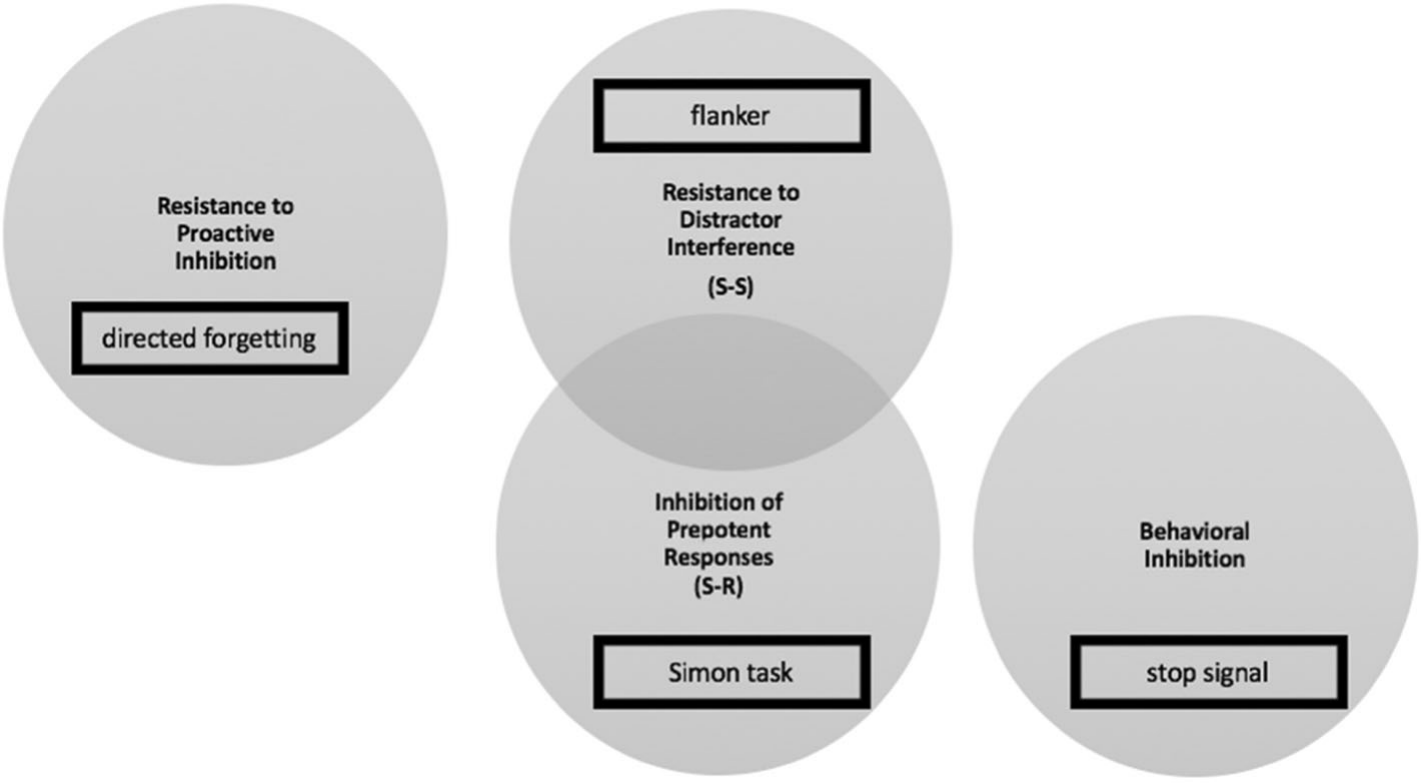
Each circle represents a hypothetical inhibition factor. Overlapping circles indicate factors that are correlated. An example task (In directed forgetting, participants first memorized two memory sets and were then instructed to ignore one set while reporting on the basis of the other set. In the stop signal, participants performed an ongoing task (e.g., a word categorization) unless the stop signal (i.e., a tone or change in color frame) occurred. In this case, they had to withhold their responses. The time between the presentation of the stimulus and the stop signal is adapted such that participants can only stop their reaction successfully on 50% of the trials.) that provides a measure of each form of inhibition is shown within the black rectangles

If the interference scores derived from any two nonverbal interference scores correlate, this can be taken as evidence that they share a conflict-resolution mechanism. Kornblum’s taxonomy implies that different mechanisms are employed to resolve S-S and S-R conflict. Thus, the intertask correlations between the interference scores for the four tasks shown in Fig. 1 should increase from pairs of tasks that share neither type of conflict (namely, a pure S-S vertical Stroop task and a pure S-R Simon task) to one type (e.g., S-R for both the Simon task and the spatial Stroop task) to both (namely, a spatial Stroop and flanker task). The overall pattern of intertask correlations provided little support for the hypothesis that nonverbal interference tasks involving the same type of incompatibility recruit a common inhibitory control mechanism (Paap, Anders-Jefferson, Mikulinsky, Masuda, & Mason, 2019). Even two versions of the same task can fail to correlate as Salthouse (2010) reported for the letter and arrow instantiations of the flanker task. However, exploring the construct of inhibition through individual differences often goes beyond zero-order intertask correlations and uses latent-variable analyses such as confirmatory factor analysis (CFA) or structural equation modeling (SEM). Figure 2 represents one perspective on these efforts. *Resistance to PI* (the circle on the left) is the ability to prohibit memory intrusions from information that was previously relevant to the task but has since become irrelevant. Latent variable analyses from Friedman and Miyake (2004) to Pettigrew and Martin (2014) to Stahl et al. (2014) have concluded that *Resistance to PI* should be interpreted as a separate factor.

The locus of greatest controversy (represented by the overlap in the middle circles) is whether *Resistance to Distractor Interference* (S-S conflict resolution) is separable from *Inhibition of Prepotent Responses* (S-R conflict resolution). The former refers to a control process that reduces the competition between representations of the task relevant and irrelevant information, while the latter refers to a control process that reduces the competition between competing responses. Several latent variable analyses have shown good fits for models that assumed that *Resistance to Distractor Interference* and *Inhibition of Prepotent Responses* are correlated but distinct forms of inhibition (Friedman & Miyake, 2004; Stahl et al., 2014). However, this conclusion has been challenged by Rey-Mermet, Gade, and Oberauer (2018) in a study requiring each participant to complete six tasks assumed to reflect *Inhibition of Prepotent Responses* and five assumed to reflect *Resistance to Distraction*. Rather than examining only the fit of various models, Bayesian hypothesis testing was also conducted. These additional tests showed that the data provide ambiguous evidence as to whether there is one inhibition factor or two; or, if two, whether they are correlated or orthogonal. Another problem pointed out by Rey-Mermet et al., both in their data and other latent-variable analyses, is that for each latent variable, one loading was substantially higher than the others (e.g., the interference score for the number Stroop task^2^ for *Resistance to Distraction*). Consequently, each latent variable represents mainly the variance of one task, with the remaining tasks saddled with high error variances. These problems led Rey-Mermet et al. to suggest that, regardless of which model fits the data better, all models had poor explanatory power. As shown in Fig. 2, Stahl et al. found strong evidence that *Behavioral Inhibition* (based on performance in stop-signal, go/no-go,^3^ and antisaccade tasks^4^) should be interpreted as a separate factor, but all three of these tasks were used in Friedman and Miyake’s (2004) seminal study to define *Prepotent Response Inhibition*! From their view of this evidence, Rey-Mermet et al. suggested that the nonverbal tasks used to assess “inhibition” do not measure a common underlying construct but instead measure the highly task-specific ability to resolve the interference arising in each task. For them the “… inevitable implication is that studies using a single laboratory paradigm for assessing or investigating inhibition do not warrant generalization beyond the specific paradigm studied*”* (p. 515).

Of course, a middle ground exists between inhibition as a unitary construct and the conclusion that conflict resolution is always task specific. In a companion article to this one, Paap et al. (2019) reported that, contrary to the pattern predicted by Kornblum’s taxonomy, an exploratory factor analysis of the four nonverbal interference tasks yielded a coherent cluster of tasks when the conflict was between two dimensions of the target stimulus (Simon, spatial Stroop, and vertical Stroop tasks) that did not correlate with the flanker task where the conflict is along the same dimension of different stimuli. Many theorists have suggested that conflict in the flanker task is resolved by spatially attending to the target stimulus (e.g., Magen & Cohen’s Dimension Action model, 2007). If spatial attention is construed as a filter or the upregulation of task-relevant information, then it clearly contrasts with inhibition of irrelevant and competing representations.

## Are interference scores (or executive functions more broadly) enhanced by practice?

One major purpose of this article was to investigate the potential relationships between highly practiced skills (e.g., bilingualism, video gaming, music performance, and /mindfulness/meditation) and the interference scores derived from the four nonverbal interference task shown in Fig. 1 that were completed by 201 participants. Many activities and skills seem to require good inhibitory control. Does the ubiquitous practice required to become skilled in a specific domain (generically dubbed X) enhance a general inhibitory control ability that would transfer to other domains? It would seem that a good test of this possibility would be to show a positive relationship between the time spent doing X, or the proficiency in X, or the amount of training in X, and performance in a task that no participants have practiced, requires little declarative knowledge, and that transparently measures inhibitory control. This, one might argue, accounts for the popularity of the flanker, Simon, and spatial Stroop tasks.

Before reviewing the relevant research literature for each of the five activities, clarification of the distinction between inhibitory control and executive functions (EF) is important. EFs consist of a set of general-purpose control processes that are central to the self-regulation of thoughts and behaviors and that are instrumental to accomplishing goals. Research on EF has often focused on the three components initially identified by Miyake et al. (2000) using latent variable analyses: updating, switching, and inhibitory control. Inhibitory control was inferred from performance measures in three different tasks that all involve competition and therefore require some type of conflict resolution such as the inhibition of a prepotent response. Likewise, a general switching ability was inferred from performance on three different tasks that frequently required participants to switch from one task (e.g., judgments about color) to another (e.g., judgments about shape). The third latent variable— updating of working memory representations—requires monitoring and coding incoming information for task relevance and then appropriately revising the information held in working memory. In Miyake et al. (2000), each of three observed measures significantly loaded on the expected latent variable, establishing that these three EFs can be considered as separate abilities. Furthermore, at the higher level of the analysis, the three latent variables also correlated with one another, and this is consistent with the assumption that the latent variables are components of a common EF ability.

Miyake and Friedman (2012) now favor a variation on the simple hierarchical model described above. They compared the fit of the simple hierarchical model to a more complex second-order (“nested”) model, where the nine observed measures are allowed to load on common EF, and the three latent variables compete in accounting for the remaining variance. The best solution for the second-order model resulted in all nine measures loading on the common EF and with only two of the nested components (updating and switching) still making unique contributions. Putting this together, the model supports a theory of a general EF ability with separate updating and switching components and an inhibition component that is not separable but is moderately linked to general EF ability. This analysis led Miyake and Friedman (2012) to conclude that EF has both unity (a common EF) and diversity (additional specific abilities associated with switching and updating).

Many of the studies reviewed on the potential benefits of bilingualism, music, meditation, video gaming, and exercise were designed and interpreted within the framework of Miyake and Friedman’s earlier framework, where inhibition, switching, and updating were considered three separable components of EF. Thus, the review for each activity often starts with meta-analyses on the relationship between a specific activity/skill (e.g., music training or performance) and the evidence that it enhances general EF or an even broader set of cognitive abilities. For each activity, this initial subsection is followed by a more specific consideration of the studies that specifically tested for advantages in the “inhibition” component.

### Effects of music performance

In a mini-review, Benz, Sellaro, Hommel, and Colzato (2016) summarized evidence that music performance benefits several aspects of cognitive ability ranging from phonemic awareness to working memory. Sala and Gobet (2017) performed a more formal meta-analysis of the effects of music training on children and young adolescents’ intelligence and memory. Although the effect sizes were of moderate size (about d = .35), an inverse relation existed between the size of the effects and the methodological quality of the study design, which was indexed as the presence of an active control group and the random assignment of participants to the treatment groups. The authors conclude that music training does not reliably enhance cognitive or academic skills.

Although the Sala and Gobet meta-analysis included 38 studies that spanned 118 comparisons, it did not include tests of the hypothesis that music training enhances interference control, the focus of this paper. We turn next to the studies that do so. Bialystok and DePape (2009) are often cited as showing benefits of music performance on inhibitory control, and that study did include a spatial Stroop task like the one used in the present study. However, the musician “advantage” was in the overall speed and not in the interference scores. Estimating the interference effect from their Fig. 1, the trend actually favors the non-musicians (approximately 20 ms compared to approximately 25 ms).

A quasi-experiment by D’Souza, Moradzadeh, and Wiseheart (2018) is also relevant. The design compares four groups defined by the combinations of bilingual or not and musician or not in multiple tasks tapping into EF. The results revealed that musically trained individuals, but not bilinguals, had enhanced working memory, but neither skill enhanced inhibitory control, as reflected in flanker or Stroop interference. Similarly, Slevc, Davey, Buschkuehl, and Jaeggi (2016) reported no significant correlations between music ability (or years of lessons or practice) and either an auditory Stroop task or a spatial Stroop task similar to the one used in the present study.

In a thorough discussion, Valian (2015) opines that reverse causality is very plausible in the music domain, especially if general EF contributes to mastery or excellence. Thus, in the types of studies discussed so far, any advantages for musicians might be due to individuals with better EF being attracted to and maintaining their interest in more music training.

Only one study appears to show a benefit of music training on a form of inhibitory control. Moreno et al. (2011) showed that a fairly short-term training regime improved performance on a go/no-go task compared to a comparable active control group (e.g., visual arts training). Possible implications of this training advantage for predicting the relationship between music training and performance on the nonverbal interference tasks used in the present study are complicated because they would have to be predicated on the assumption that the go/no go tasks and nonverbal interference tasks share a common inhibition mechanism. The full set of reviewed results most closely align with the expectation of no relationship between music experience and interference control in nonverbal interference tasks.

### Effects of bilingualism

Bilinguals have been claimed to perform better than monolinguals in nonverbal interference tasks because they constantly practice inhibiting the language currently not in use. When bilinguals intend to produce an utterance in a target language, without a doubt, the translation equivalents in the other language become coactivated and create conflict (*see* Paap, 2018a for a review) that could be resolved by recruiting domain-general inhibitory control. The effects of bilingualism in the current dataset have been reported in the companion article (Paap et al., 2019). No statistically significant differences exist between bilinguals and monolinguals in any of the four tasks, nor was there a bilingual advantage in the composite interference-score.

Three recent meta-analyses converge on the conclusion that significant bilingual advantages in inhibitory control are relatively rare (15% of all comparisons), that the average effect sizes are very small, and that evidence exists for publication bias, which when taken into account, appears to completely eliminate the effect. In the meta-analysis by Paap et al. (2019), the mean bilingual advantage across all 146 comparisons was + 4.4 ms. The meta-analyses by Lehtonen et al. (2018) examined bilingual advantages across six domains of executive functioning (EF), but their analysis of inhibitory control is most central to our focus. Their meta-analysis used a wider definition of inhibitory control tasks and identified a more heterogeneous set of 212 effect sizes compared to Paap et al. (2019). The mean effect size for inhibitory control in Lehtonen et al. was Hedge’s *g* = + 0.11 [+ 0.05, + 0.18], but when corrected for bias the mean was no longer significant; that is, *g* = − 0.02 [− 0.12, + 0.08]. Donnelly, Brooks, and Homer (2019) reported a meta-analysis of 80 studies using a multiverse analysis approach where each research question was tested many times while making different decisions about the inclusion criteria. The bilingual-advantage effect size, corrected for publication bias, was negative; that is, g = −.22 [−.35, −.09].

The null results obtained with the four tasks shown in Fig. 1 and the meta-analyses led Paap et al. (2019) to conclude that the most likely reason for bilingualism not enhancing a general inhibitory control ability is that bilingual language control is encapsulated within the language processing system. That is, any conflict resolution between the two languages of a bilingual relies on language-specific rather than domain-general mechanisms.

### Video gaming

In a trio of meta-analyses, Sala, Tatlidil, and Gobet (2017) assessed the relationship between video game playing and five broad measures of cognitive ability. They reported weak correlations with continuous measures of video game skill, small (but statistically significant) differences between players and nonplayers, and negligible differences between groups assigned to video game training compared to various types of control groups. The effects were not moderated by the type of cognitive ability, but given our focus on nonverbal interference tasks, the flanker task, importantly, was assigned to one cluster (*visual attention/processing*) and the Simon task to another (*cognitive control*). The remainder of our discussion of video gaming focuses only on the flanker, Simon, and spatial Stroop tasks.

Dye, Green, and Bavelier (2009) intriguingly reported that, across four age groups ranging from 7 to 22 years old, video game players were faster and as accurate as nonplayers, but consistently showed larger flanker effects in the fish version of the ANT. The pattern of results observed by Dye et al. invites the interpretation that video game players have learned how to distribute their attention to a greater area of the visual field, an adjustment that pays off in most video games because task relevant stimuli often pop up in parafoveal or peripheral locations. In contrast, in a flanker task, paying greater attention to the flankers has minimal benefit on congruent trials and incurs a heavy cost on incongruent trials. This intriguing result was unfortunately not reproduced by Irons, Remington, and McLean (2011) when using groups at the upper age range of Dye et al.’s study and a letter version of the flanker task rather than the ANT. Two subsequent studies using the arrow version of the flanker task also showed no differences between players and nonplayers (Caine, Landau, & Shimamura, 2012; Gobet et al., 2014).

A study by Hutchinson, Barrett, Nitka, and Raynes (2016) differed from the studies described above with respect to both design and task. Instead of partitioning individuals into players and nonplayers on the basis of self-reported experience, Hutchinson et al. randomly assigned nonplayers to groups that trained on either a first-person-shooter game, a visual training game, or a no-training control. Training took place for an hour a day for 10 consecutive days. The dependent variable was the pre-post difference in the magnitude of the interference score obtained in a Simon task. Only the video game group showed a significant reduction in the Simon effect. However, Unsworth et al.’s (2015) found that continuous measures of video game play predicted neither the flanker effect (r = −.06) nor the spatial Stroop task (r = −.08).

Like Unsworth et al., the present study treats self-reported video game playing as a continuous variable and allows comparison between a Simon task, two spatial Stroop tasks, and a flanker task that are otherwise very similar to one another. Thus, the present study will help adjudicate if the Simon effects and only the Simon effects decrease as the frequency of video gaming increases.

### Effects of mindfulness/meditation

Consistent with Tang, Ma, Wang, et al.’s (2007) seminal study, participants randomly assigned to training in mindfulness/meditation often show reduced interference scores compared to those assigned to an active control group. More specifically, in Tang et al., an integrative body-mind training group showed greater pretest to post-test improvement in the interference scores of an ANT following five daily sessions lasting 20 min compared to a control receiving relaxation training. As shown in Table 1, 13 other studies have also reported statistically significant benefits of mindfulness/meditation training, eight of which used random assignment to an active control group. In contrast, 15 training studies showed no significant effects of mindfulness/meditation on RT interference scores.

**Table 1:** Results of studies testing for benefits of mindfulnessmeditation on interference control

Study	Task	Duration.	# in exp.	# in control	Ran-dom	Active?	Decision
Training studies comparing trainees to controls							
Anderson, Lau, Segal, and Bishop (2007)	color Stroop	8 weeks 2 h/week	39	33	yes	waitlist	ns
Colzato, Sellaro, Samara, and Hommel (2015)	Simon	17-min session	18	18	yes	open attention	ns
Esch et al. (2017)	ANT	5 days 1.5 h/day	16	15	yes	no	ns
Larson, Steffen, and Primosch (2013)	arrow flanker	14.5 min	28	27	yes	relaxation	ns
Lai, MacNeil, and Frewen (2014)	ANT	15 min	23	21	yes	counting	ns
Lim and Qu (2016)	ANT	15 min	41	39	yes	dance, sing, count	ns
Norris, Creem, Hendler, and Kober (2018), Study 1)	arrow flanker	10 min	18	19	yes	reading	ns
Norris et al. (2018), Study 2)	ANT	10 min	29	27	yes	reading	ns
Oken et al. (2016)	flanker	6 weeks 60–90 min/wk	60	68	yes	waitlist	ns
Oken et al. (2016)	color Stroop	6 weeks 60–90 min/wk	60	68	yes	waitlist	ns
Polak (2009)	ANT	2 sessions 15 min each	50	50	yes	relaxation	ns
Polak (2009)	color Stroop	2 sessions, 15 min each	50	50	yes	relaxation	ns
van den Hurk et al. (2012)	ANT	8 sessions, 2.5 h each	34	37	yes	waitlist	ns
Wahbeh, Goodrich, Goy, and Oken (2016)	ANT	8 weeks + 20 min of daily homework	27	25	yes	sitting quietly	ns
Jha, Krompinger, and Baime (2007)	ANT	6 weeks, 3 h/class	17	17	no	waitlist	ns
Ainsworth, Eddershaw, Meron, Baldwin, and Garner (2013)	ANT	3 days, 1 h/day focused att.	24	24	yes	relaxation	sig
Ainsworth et al. (2013)	ANT	3 days, 1 h/day openness att.	25	24	yes	relaxation	sig
Allen et al. (2012)	number Stroop	8 sessions, 2 h each	19	19	yes	selective listening	sig
Becerra, Dandrade, and Harms (2017)	ANT	8 weeks, 24 min per wk	23	23	yes	waitlist	sig
Elliott, Wallace, and Giesbrecht (2014)	ANT	week long retreat, 3–4 h/day	22	19	yes	waitlist	sig
Fan, Tang, Tang, and Posner (2014)	Stroop color	5 days, 20 min/day	21	22	yes	relaxation	sig
Felver, Tipsord, Morris, Racer, and Dishion (2017)	ANT	8 weeks, 90 min./week	24	23	yes	waitlist	sig
Moore, Gruber, Derose, and Malinowski (2012)	Stroop color	16 weeks, 10 min/week	12	16	yes	waitlist	sig
Quan, Wang, Chu, and Zhou (2018)	ANT	7 days, 100 min/day	24	24	yes	relaxation	sig
Tang et al. (2007)	ANT	5 days, 20 min/day	24	24	yes	relaxation	sig
Tang (2009)	ANT	4 weeks, 11 h total			yes	relaxation	sig
Wenk-Sormaz (2005)	color Stroop	20 min,	20	20	yes	leaning	sig
Baijal, Jha, Kiyonaga, Singh, and Srinivasan (2011)	ANT	1 to 2 years	79	76	no		sig
Study	Task	Experience	# in exp.	# in control	Ran-dom	Controls	Decision
Cross-section studies comparing meditators to non-meditators							
Andreu et al. (2017)	letter flanker	5 years	31	30	no	athletes	ns
Jo, Malinowski, and Schmidt (2017)	ANT	13.1 years	22	23	no		ns
Isbel and Mahar (2015)	ANT	at least 6 months	23	21	no		ns
Otten et al. (2015)	ANT	min of 3 years	22	22	no		ns
Schotz et al. (2015)	flanker	min of 3 years	20	20	no		ns
Wei, Dong, Yang, Luo, and Zuo (2015)	ANT	14.6 years	18	22	no		ns
Wittmann et al. (2015)	ANT	10 years	42	42	no		ns
Moore and Malinowski (2009)	color Stroop	> 6 weeks	25	25	no		sig
Sperduti, Makowski, and Piolino (2016)	ANT	25.5 years	16	16	no		sig
Jha et al. (2007)	ANT	?	?	17	no		sig
van den Hurk, Giommi, Gielen, Speckens, and Barendregt (2010)	ANT	14.5 years	20	20	no		sig

Note. *ns* statistical test was not significant, *sig* statistical test was significant

Listed in the lower section of Table 1 are characteristics and outcomes of studies comparing the interference scores of experienced meditators to a non-meditator control group. Studies comparing two naturally occurring groups are, of course, vulnerable to confounding factors that can either generate false differences or cancel out genuine ones, but these designs do have the advantage of using individuals who have meditated for many years and/or who engage in frequent meditation. Given that this second set of studies employs a much bigger “dose” of meditation, only 4 of the 12 studies showing a significant treatment effect is surprising. The present study will take advantage of the natural variation in mindfulness/meditation experiences across a large sample of college students to assess the correlation between mindfulness/meditation and the interference scores across the four nonverbal interference tasks. As shown in Table 1, most of the previous tests of the effects of mindfulness/meditation on interference scores have been limited to the flanker and its ANT hybrid.

### Effects of physical exercise

Does exercise enhance EF? If yes, how does mode, frequency, duration, and intensity moderate the relationship? Drawing on their own meta-analysis, Diamond and Ling’s (2016) concluded that aerobic exercise or resistance training without a cognitive component produces little or no EF benefit. Stated more poetically, EF benefits from mindful but not mindless physical activity. Hillman, McAuley, Erickson, Liu-Ambrose, and Kramer (2019) respectfully, but forcibly, disagreed with the mindful, but not mindless conclusion, as they believed Diamond and Ling’s meta-analysis failed to consider all of the relevant articles, that the results of some were misinterpreted, and that some of the interventions were mischaracterized. Part of the evidence reviewed by Hillman et al. supporting the benefits of exercise was a recent meta-analysis by Northey, Cherbuin, Pumpa, Smee, and Rattray (2017). This meta-analysis included 333 effect sizes drawn from 36 studies. Each effect was based on a randomized control trial of physical exercise interventions in adults older than 50 years with an outcome measure of EF. The mean effect size was *d* = .29 with 95% CI of .17 to .41, *p* < .01. The moderator analyses showed greater benefits when the frequency of exercise was high (5–7 times per week), of at least medium duration (45 to 60 min) and at least of moderate intensity.

The Northey et al. meta-analysis only provides an analysis of general EF and does not provide specific information about inhibitory control or the interference scores derived from nonverbal interference tasks. Specific examinations of the relationship between physical exercise and interference scores are mixed, as illustrated in Themanson and Hillman’s (2006) study using a version of the flanker task. This study is unusual in that it tests for effects of both acute (30 min of treadmill versus rest) and chronic exercise (high versus low levels of cardiorespiratory fitness). Although the highly fit group exhibited reduced error-related negativity, increased error-related positivity, and increased post-error response slowing compared to the less fit group, of critical interest to our focus, no group differences existed in the magnitude of the flanker effect. The acute exercise manipulation did not affect any of the dependent measures, which was in contrast to Chang, Pesce, Chiang, Kuo, and Fong (2015), who reported smaller flanker effects for fit young adults following 40 min of cycling (at levels causing heart rates at 70–85% of individual maximums) in comparison to a control group that read an exercise-related book.

Our previous work and the present study focuses on the frequency of exercise and uses this item: “How often in a typical week do you exercise, work out, or participate in a sport?” This probe was never significantly related to either Simon or flanker effects (e.g., Paap & Greenberg, 2013; Paap, Johnson, & Sawi, 2014). In a recent study investigating the relationship between autistic tendencies and exercise (Mason et al., 2018), 196 young adult college students responded to the exercise probe and also completed a spatial Stroop task. The correlation between frequency of exercise and the interference score was close to zero: r = +.005. A related item emphasized ability and not frequency of participation: “Team sports often involve dividing your attention between a ball, a goal, your opponents, and your teammates. Do you excel at these sports?” The correlation between this item and the spatial Simon effect was also minimal: r = −.049.

### Summary across five activities

For each activity, researchers have hypothesized that some form of cognitive control is required and that engaging in the activity and developing the skill leads to an enhancement of domain-general control. However, in each case, the previous results are inconclusive regarding the presence or absence of a relationship with a latent variable for inhibition or any of the individual nonverbal interference tasks.

## Other individual differences assumed to be related to inhibitory control

### General fluid intelligence

The concept of “general intelligence” or Spearman’s g (Spearman, 1927) is basic to the study of individual differences. Any set of tasks purported to measure cognitive abilities is likely to be peppered with positive correlations. One interpretation of a plethora of weak and positive correlations is that some general or *g* factor makes some contribution to success in a variety of tasks. This framework led to several standard IQ tests such as the Wechsler Adult Intelligence Scale (WAIS; Wechsler, 1955). Tests that involve novel problem-solving such as Raven’s Progressive Matrices (Raven, Court, & Raven, 1977) or Cattell’s Culture Fair Test (Cattell, 1971) target general “fluid” intelligence (gF) that does not require acquired knowledge or, as it sometimes referred to, “crystallized intelligence.” An inviting hypothesis is that gF contributes to tasks designed to test EF and/or its inhibitory control component. For example, Duncan, Emslie, Williams, Johnson, and Freer (1996) assume that, in large part, gF reflects the EFs in the brain’s frontal lobes that, when impaired, result in goal neglect (even in circumstances where the correct action is understood and has not been forgotten).

The possible relationship between latent variables for gF and EF has been intensely studied and debated. Engle, Kane, and their collaborators (e.g., Engle & Kane, 2004; Kane, Conway, Hambrick, & Engle, 2007) have advanced a theory that hypothesizes that EF (executive attention, EA, is their preferred term) is a basic cognitive ability that drives individual differences in both gF and working memory capacity (WMC). In short, these authors assume that WMC and gF rely on the effective focusing of attention on task-relevant information and on the blocking of potential distraction. As many of their earlier structural models used the antisaccade, flanker, and Stroop color-word tasks to derive their EA factor, this work provides consistent evidence that their EA factor is strongly related to a gF factor. However, notably, the EA factor was dominated by the antisaccade task, and the flanker task loaded rather weakly on the EA factor.

Friedman and Miyake’s (2016) review concludes that the relationship is more complicated. First, their preferred *unity and diversity* model does not include a separable inhibition factor because (across multiple independent datasets) they were not able to extract an inhibition factor because the Common EF factor explained all the correlations among the inhibiting tasks. One possible interpretation is that the Common EF factor is inhibition or that inhibition is most central to all EFs (e.g., Valian, 2015). On the other hand, it has also been interpreted as evidence that there is nothing special about inhibition (Banich & Depue, 2015). But the possibility most relevant to this section is the hypothesis that Common EF is Spearman’s *g*. However, when Friedman et al. (2006, 2008) controlled for the correlations between factors, only updating was significantly related to intelligence. Likewise, Chuderski, Taraday, Necka, and Smolen (2012) reported that their EA factor strongly correlated with a storage capacity factor, and when capacity was entered as a gF predictor, the link between EA and gF disappeared.

Rey-Mermet, Gade, Souza, von Bastian, and Oberauer (2019) present two additional challenges to the hypothesis that inhibitory control is related to gF. First, despite using adaptive versions of standard interference tasks (e.g., Stroop, arrow flanker, and Simon) that enabled the use of accuracy measures with adequate test reliabilities, the six tasks could not form a coherent latent variable. Second, the regression coefficients between each of the six separate measures of inhibition and the latent variable for gF were all small and nonsignificant. Most germane to our study, the coefficients for the arrow flanker and Simon measures were − .10 and + .08, respectively.

Turning to specific tests of the relationship between measures of gF and nonverbal interference that were not part of a latent factor analysis, the evidence is inconsistent. Significant correlations have, for example, been reported for the Simon task (r = −.23, Paap & Greenberg, 2013) and flanker task (r = −.33, Unsworth & Spillers, 2010; r = −.30; Chen et al., 2019) but are often nonsignificant for similar instantiations of these tasks: Simon (Rosselli, Ardila, Lalwani, & Velez-Uribe, 2015), flanker (Bialystok & Barac, 2012; Blom, Boerma, Bosma, Cornips, & Evaraert, 2017; Paap & Greenberg, 2013; Unsworth, Spillers, & Brewer, 2009), and spatial Stroop (Mercier, Pivneva, & Titone, 2014; Paap, Anders-Jefferson, Mason, Alvarado, & Zimiga, 2018). In summary, the existing link between interference scores and gF appears to be weak and inconsistent.

### Sex

The possibility of sex differences in “inhibitory control” as measured in nonverbal interference tasks is surprisingly understudied. Two studies using spatial Stroop tasks similar to ours reported statistically significant male advantages in the form of smaller interference effects. Stoet (2016) tested 236 males and 182 women in an online study and reported 42 ms interference scores for males and 29 ms for females. Evans and Hampson (2015) tested 90 males and 86 females and, estimating from their Fig. 4, it appears that the interference effects were approximately 60 ms and 40 ms, respectively.

### SES

SES can impact the development of higher order cognition. For example, Mezzacappa (2004) had 249 6-year-old children complete a version of the flanker task with the arrows replaced by hungry fish. Socially advantaged children exhibited smaller interference effects in both RT and accuracy compared to their less advantaged peers. However, in the population that is the focus of this study—young adult university students—various measures of SES yield no significant correlations with interference scores (e.g., Antón, Carreiras, & Duñabeitia, 2019; Paap et al., 2014; Paap & Greenberg, 2013). If interference scores did reflect general inhibitory control, then one might advance the conjecture that most college students who come from families with low SES benefit from countervailing opportunities and experiences that enable the development of EF and supported the pathway to higher education.

## The relationship between self-control, impulsivity, and interference scores

### Self-control

An appealing conceptual definition of *self-control*, borrowed from Baumeister, Vohs, and Tice (2007) and Duckworth and Kern (2011), defines self-control as the capacity to alter one’s own actions, especially to bring them into line with personally valued goals and standards. However, as Duckworth and Kern observed, extraordinary diversity exists in how the construct of self-control is operationalized. One reflection of that diversity is the plethora of alternative terms: self-regulation, self-discipline, willpower, effortful control, ego strength, and inhibitory control, among others. Duckworth and Kern conducted a meta-analysis of 282 samples to examine the evidence for convergent validity both between and within several types of self-control measures. A major purpose of the present study is to further examine the relationship between self-report scales of both self-control and impulsivity and the interference effects in the four nonverbal interference tasks illustrated in Fig. 1. Duckworth and Kern considered a broad set of EF tasks that included the Stroop and flanker but also other classic tasks such as switching, stop-signal, Go/No-go, tower-of-London,^5^ and Trails.^6^ The correlation between each of these EF tasks and measures based on self-reports tended to be quite low (ranging from r = −.02 to r = +.18). The correlation between self-report of self-control and the Stroop task was r = +.12, but most of these studies likely used Stroop’s original color-word version, while relatively few, if any, used a nonverbal spatial Stroop task. More recent findings follow the same pattern. For example, Allom, Panetta, Mullan, and Hagger (2016) show near-zero correlations between a composite measure of self-control and measures of EF derived from a stop-signal and Stroop color-word interference task. Thus, the present study fills a void because the relationship between the congruency effects (RT incongruent minus RT congruent) obtained in nonverbal interference tasks and trait measures of self-control and impulsivity have not been examined.

### Impulsivity

Spontaneous behaviors that are triggered by internal or external stimuli or by response tendencies that are incompatible with long-term goals and well-being are often called impulsive. Following tradition, we consider self-control and impulsivity as separate traits that are positive and negative, respectively. They are, however, operationalized with similar items and, as reported in the results, the correlations between self-reported measures of impulsivity and self-control are quite strong.

The narrative of the Stahl et al. (2014) study discussed earlier in the context of the fractionation of the inhibition construct framed their SEM model as an investigation of impulsive behavior. Their six-factor SEM model used 16 cognitive measures to identify six components of impulsivity. No evidence was found for a relation between the behavioral impulsivity factors (cognitive tasks) and self-reported impulsivity. Ironically, the 16 measures selected to load on the six factors of impulsivity did not include the flanker effect (although all participants did complete a flanker task). Thus, we cannot be certain that self-reported impulsivity was also unrelated to the flanker effects.

Jauregi, Kessler, and Hassel (2018) examined the relationship between several self-rated measures of impulsivity and various cognitive tasks that plausibly should reflect impulsive behavior. None of the cognitive tasks involved interference scores, but the go/no-go and stop-signal results that are often associated with behavioral inhibition (*see* Fig. 2) failed to correlate with any of the self-report measures. Likewise, neither Reynolds, Ortengren, Richards, and de Wit (2006) nor Aichert et al. (2012) found significant correlations between several measures of impulsivity and behavioral inhibition as measured using the go/no-go and stop-signal tasks. In contrast, Wilbertz et al. (2014) reported that the UPPS subdomain of Urgency did predict stop-signal scores. Similarly, Malesza and Ostaszewski (2016) found a significant correlation (r = 1.74, *p* < .05) between scores on the Barratt Impulsiveness Scale (11th revision; BIS-11) (Patton, Stanford, & Barratt, 1995) and performance in the stop-signal task. In summary, investigations of the relationship between self-rated impulsivity and cognitive tasks assumed to reflect inhibition have been dominated by studies using the stop-signal and go-no go tasks.

Most central to our focus on nonverbal interference scores, Enticott, Ogloff, and Bradshaw (2006) reported a significant correlation (*r* = .55, *p* = .001) between BIS-11 scores and spatial Stroop effects from a task similar to that illustrated in Fig. 1. However, Aichert et al. (2012) reported no significant correlations between impulsivity and Stroop color-word interference. The design of the present study affords an opportunity to see if the correlation between self-rated impulsivity and spatial Stroop interference reported by Enticott et al. (2006) can be replicated in a considerably larger sample and considers whether it generalizes to three other nonverbal interference tasks.

### Research questions

Paap et al. (2019) reported, for this dataset of 201 participants, that the two spatial Stroop tasks and the Simon task cohere as a latent variable that excludes the flanker effect. This clustering is inconsistent with both predictions from Kornblum’s taxonomy and with the hypothesis that *Resistance to Distractor Interference* (S-S conflict resolution) is separable from *Inhibition of Prepotent Responses* (S-R conflict resolution). They also tested and failed to find evidence for the hypothesis of bilingual advantages in any type of interference costs. This report extends the investigation of the enhancement of general inhibitory control through practice of activities that seemingly require control from bilingualism to music performance, video gaming, and mindfulness /meditation. A second purpose is to test if the individual attributes of sex, gF, age, immigrant status, and SES predict interference scores. A third purpose is to determine if self-reported measures of trait impulsivity or self-control predict interference scores as they should if both measure the same construct.

## Methods

### Sequence of events

All parts of the study were conducted in a single session of at least 60 min. The first activity was to obtain written consent to participate using a form approved by the SFSU IRB. This was followed by (1) the four nonverbal interference tasks, (2) the Raven’s test, (3) the language background questionnaire, (4) demographic questions, (5) questions about special experiences, (6) the impulsivity and self-control scales, and (7) the Mulitilingual Naming Task (MINT) (Gollan, Weissberger, Runnqvist, Montoya, & Cera, 2011).

### Participants

The participants were 213 SFSU undergraduate students who either received extra credit or chose participation as one option for a class research assignment. Their mean age was 23.7 years. Nine participants failed to complete all parts, and their incomplete data were not included in any analyses. Of the remaining 204 participants, three were eliminated for performance reasons on the nonverbal interference tasks. The data from one participant were excluded because the overall proportion correct (0.84) was more than 6.7 standard deviations below the mean of 0.97. The data from two other participants were excluded because their overall mean RT was more than a 1000 ms and more than 7 standard deviations above the grand mean of 473 ms. The final set of 201 participants included 149 females and 52 males.

### Trial definition for all tasks

The four tasks were described in the introduction with reference to Fig. 1. The protocol was programmed in DirectRT. Each trial was initiated with a plus sign in the center of the display for 500 ms that served as a fixation point and warning signal. The plus sign was followed by the imperative stimulus (row of arrows for the flanker and a single arrow for the other tasks) that remained in view until a valid response was made. Any response longer than 2 s was followed by the prompt “please try to respond faster!” Incorrect responses were followed by a “beep.” The fixation point for the next trial appeared immediately after the participant responded. Thus, the response stimulus interval was 500 ms.

### Display dimensions

Each arrow regardless of its location or direction was 7.5 cm (8.1°) in length and 5.4 cm (5.8°) in maximum width. The gap between the center fixation and the nearest edge of a horizontally displaced horizontal arrow (or a vertically displaced vertical arrow) was 4.5 cm (4.9°). The gap between the center fixation and a horizontally displaced vertical arrow (or a vertically displaced horizontal arrow) was 5.75 cm (6.2°). The gap between adjacent arrows in the flanker task was 2.54 cm (2.7°) The visual angles shown in parentheses assume a viewing distance of 53 cm.

### Design

Each task started with a practice block of 20 trials where the imperative arrow was centered at fixation. Practice was followed by an experimental block of 160 trials. Half the trials required pressing the left key and half the right key. However, 75% (120 trials) of the trials were congruent compared to only 25% (40 trials) that were incongruent. Making incongruent trials less frequent usually increases the interference scores. The order of the four tasks was counterbalanced across participants using a Latin square, whereby each task appears an equal number of times in each position and is preceded by and followed by each of the other three tasks an equal number of times.

### The predictor variables

#### Bilingualism

Extensive information was solicited from the participants about their exposure to and use of English and other languages and is reported in detail in Paap et al. (2019). For each language an individual was exposed to, they were asked to rate, separately, their speaking, listening, reading, and writing proficiency on an eight-point scale ranging from 0 (*no exposure to another language*) to 7 (*Super Fluency: Better than a Typical Native Speaker)*. The convention was adopted to use L1 to refer to the language with the highest rated proficiency regardless of whether it was English or not or whether it was a native language or not. L2 refers to the language with the next highest proficiency and so forth. When the effects of bilingualism are assessed in regression analyses, the predictor is the L2/L1 proficiency ratio.

#### Raven’s scores

Fluid intelligence was assessed using Set 1 of the Ravens Advanced Progressive Matrices (Raven et al., 1977). The task consisted of 12 items. Each item was composed of a pattern with a missing piece in the lower right. Participants were instructed to *“Look at the pattern, think what the missing part must be like to complete the pattern* correctly, both across the rows and down the columns.” Participants selected from a set of eight alternatives. The task was computerized and controlled by DirectRT. Participants were given a maximum of 2 min to respond to each item. Most responses, regardless of correctness, in this self-paced computer-controlled version were made well within the deadline. The manual states that with self-pacing, Set 1 can be used as a short 10-min test.

#### Trait impulsivity

Three of the UPPS Impulsive-Behavior subscales developed by Whiteside and Lynam (2001) were included: (lack of) premeditation, urgency, and (lack of) perseverance. Their fourth facet, sensation seeking, was not included because it seems least related to any type of cognitive control needed to perform well in nonverbal interference tasks. It also showed the weakest correlations with the other three facets (Whiteside & Lynam, 2001) and with a variety of measures of EF (Duckworth & Kern, 2011). The urgency subscale consists of 12 items, for example, *When I am upset I often act without thinking.* High scorers on urgency are likely to engage in impulsive behaviors in order to alleviate negative emotions despite the long-term harmful consequences of those actions. The (lack of) premeditation subscale consists of 11 items, for example, *I usually think carefully before doing anything*. Low scorers are thoughtful and deliberative, whereas high scorers act on the spur of the moment and without regard for the consequences. The (lack of) perseverance facet has 10 items, for example, *I finish what I start*. Low scorers can remain focused on a task that may be boring or difficult.

Whiteside and Lynam (2001) interpreted their four distinct factors as “discrete psychological processes that lead to impulsive-like behaviors” (p. 685). This led Duckworth and Kern (2011) to expect their meta-analysis to show stronger correlations within each of the four facets than the correlations across facets. Although the three subscales selected for the present study did not consistently differ from one another in the Duckworth and Kern (2011) meta-analysis, possibly, the facets will differ in the strength of their association to nonverbal interference scores.

#### Trait self-control

The Brief Self-Control Scale (BSCS; Tangney, Baumeister, & Boone, 2004) was also used to assess participants’ self-evaluations of trait self-control. The BSCS is among the most widely used questionnaires in self-control research and has been shown to predict a wide range of important outcomes including both desired and undesired behaviors (de Ridder, Lensvelt-Mulders, Finkenauer, Stok, & Baumeister, 2012; Duckworth & Kern, 2011; Tangney et al., 2004). Higher levels of trait self-control are indicated by higher scores on the BSCS. The 13 items in the BSCS seem to cut across the urgency, perseverance, and premeditation subscales developed by Whiteside and Lyman: *I am very good at resisting temptation*—urgency; *I am able to work efficiently toward long-term goals—*perseverance; and *I often act without thinking through all of the alternatives—*premeditation.

#### Single-item measures of special experiences

Our earlier work testing for the effects of bilingualism on the development and maintenance of EF included a number of single-item probes that were primarily included to ensure that the language groups were not confounded with other factors that were often assumed to enhance EF. Those that produced at least small bivariate correlations in our earlier work were included in this study and are shown in Table 2.

**Table 2:** Single-item questions about special experiences

How often do you play video games that require you to attend to many things at the same time and make fast appropriate responses? *(1) never, (2) rarely, (3) sometimes, (4) quite often (5) very often*	
How many years of musical training have you had?	
How often do you play a musical instrument? *(1) never, (2) rarely, (3) sometimes, (4) quite often (5) very often*	
How often in a typical day do you engage in two or more tasks at the same time (multitask)? *(1) never, (2) rarely, (3) sometimes, (4) quite often (5) very often*	
How often in a typical week do you exercise, work out, or participate in a sport? *(1) never, (2) rarely, (3) sometimes, (4) quite often (5) very often*	
How often in a typical week do you meditate or practice mindfulness? *(1) never, (2) rarely, (3) sometimes, (4) quite often (5) very often*	
Team sports often involve dividing your attention between a ball a goal, your opponents, and your teammates. Do you excel at these sports? *(1) not at all, (2) I am below average, (3) I am average, (4) I am better than average, (5) I am significantly better than average*	

#### Demographic items

The background questions shown in Table 3 were also tested as predictors of interference control.

**Table 3:** Background questions

What sex were you assigned at birth?	
What is your current sex?	
What is your age?	
What country were you born in? (Used to infer immigrant status.)	
Which of the following best describes the highest educational level obtained by your mother? *(1) no formal education, (2) less than 8th grade education, (3) did not graduate from high school, (4) graduated from high school, (5) attended college, but did not earn a degree, (6) earned an associate of arts degree, (7) earned a bachelor’s degree, (8) earned a graduate or professional degree that required additional education beyond a bachelor’s degree*	
Which of the following best describes the highest educational level obtained by your father? *(1) no formal education, (2) less than 8th grade education, (3) did not graduate from high school, (4) graduate from high school, (5) attended college, but did not earn a degree, (6) earned an associate of arts degree, (7) earned a bachelor’s degree, (8) earned a graduate or professional degree that required additional education beyond a bachelor’s degree*	
Relative to other families in the country where I grew up, my family’s income would be considered: *(1) very low, (2) low, (3) medium, (4) medium high, (5) high*	

## Results

### RT trimming and accuracy

Consistent with Blumenfeld and Marian’s (2014) procedure in a study using the same Simon and spatial Stroop tasks as the present study, RTs less than 200 ms or more than 2.5 standard deviations above the participant’s mean were removed. Four anticipatory responses were less than 200 ms, and 2.5% of the correct RTs were removed for being too long.

The overall mean proportion correct (PC) across the four nonverbal interference tasks was .950. All four tasks showed robust and significant congruency effects, but given the very high levels of accuracy, only the RT measures were used in the subsequent analyses. Analyses were conducted on both PCs and efficiency scores (the RT/PC ratio), but none of these analyses qualify the conclusions based on RT. Consequently, only RT analyses are reported.

### Interference scores from the four nonverbal interference tasks

Interference scores were computed for each of the 201 participants as the mean correct RT on incongruent trials minus the mean on congruent trials. The mean and standard deviations of the interference scores for the four tasks were 71.2 ms (74.6) for the flanker task, 76.8 ms (29.3) for the vertical Stroop task, 89.1 ms (41.7) for the spatial Stroop task, and 91.0 ms (38.0) for the Simon task. Although the means differ, *F*(3, 600) = 9.08, *p* < .001, the important result for present purposes is that all four tasks yield robust interference scores. However, the interference scores across the four tasks do differ with respect to their split-half (based on means computed from odd versus even trials) reliabilities as adjusted by the Spearman-Brown prophecy formula: vertical Stroop (SBP = .56), Simon (SBP = .68), spatial Stroop (SBP = .81), and flanker (SBP = .91). The lower reliabilities for the vertical Stroop and Simon tasks constrain the intertask correlations (*see* Paap & Sawi, 2016), but the exploratory factor analysis reported for this data in Paap et al. (2019) showed that the interference scores for three of the tasks did load on a latent variable: spatial Stroop (load = .59), vertical Stroop (load = .61), and Simon (load = .58).

### Which experiences, abilities, and demographics predict interference scores?

To explore the factors that have been hypothesized to be related to inhibitory control, a composite interference score was formed by taking the mean of the standardized RT interference scores for the three tasks that formed a latent variable (i.e., the Simon, spatial Stroop, and vertical Stroop tasks). A stepwise regression included the 11 predictors shown in Table 6. Characteristics of the distribution of each of these variables are shown in Table 7 of Appendix. The resulting model included two predictors (Raven’s and sex), with R = .429 showing that this model accounts for 18.4% of the variance in the composite interference effects. The standardized regression coefficients for the two predictors in this model and for the nine excluded variables are shown in Table 4 in descending order. With respect to the final model, increases in Raven’s are associated with decreases in the composite interference scores and males have smaller interference scores than females.

**Table 4:** Correlations and standardized regression coefficients (Beta) for 11 predictors of a composite interference score based on the standardized RT interference scores for the Simon, spatial Stroop, and vertical Stroop tasks

Variable	Zero-Order	Partial	Beta	*t*	*p*
1. Raven’s scores	−.377^a^	−.345	−.338	−5.11	.000
2. Sex assigned at birth	−.272^a^	−.222	−.209	−3.16	.002
3. Team sports ability	−.192^a^	−.117	−.111	−1.63	.105
4. Frequency of physical exercise	+.102	+.112	+.103	+ 1.56	.121
5. Frequency of meditation or mindfulness	+.049	+.075	+.069	+ 1.05	.296
6. Immigrant status	+.116	+.069	+.063	+ 0.96	.337
7. Frequency of playing video games	−.207^a^	−.048	−.050	−.671	.503
8. Age	+.048	+.021	+.019	+ 0.29	.769
9. L2/L1 Ratio	+.092	+.017	+.015	+ 0.23	.816
10. Years musical training	−.041	+.009	+.008	+ 0.13	.900
11. SES	−.087	+.007	+.007	+ 0.10	.922

^a^Correlation is significant at the .05 level (two-tailed)

No issues regarding collinearity were observed. When all 11 predictors are forced into the regression on the composite interference scores, the tolerance scores range from .694 to .871, and the VIF scores from 1.06 to 1.44. Using Field’s (2018) guidelines, the tolerance statistics are all well above .2, and the VIF values are well below 10. Regarding the final model, the variance proportions are .92 and .01 for Sex and Raven’s on one eigenvalue dimension and .01 and .98 on the other. The bivariate correlation matrix for the set of 15 variables (11 predictors and interference scores for each of the four tasks) is shown in Table 8 of Appendix.

**Fig. 3:**
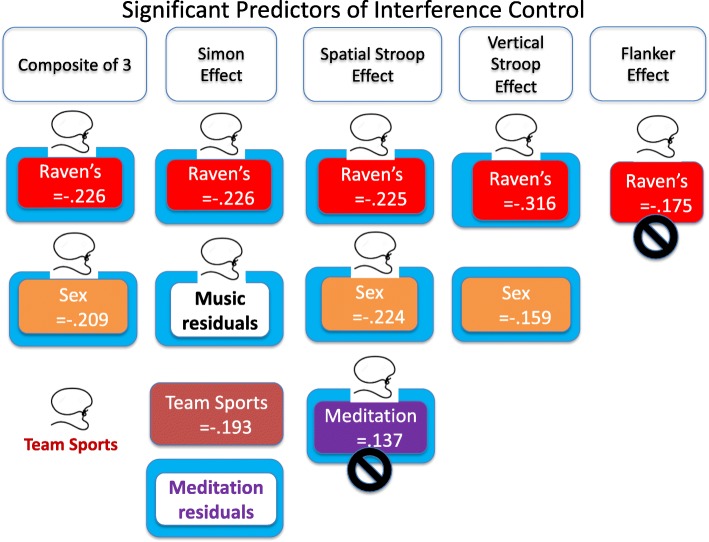
Embedded colored rectangles are the significant predictors with their beta coefficients for the stepwise regression analyses on the interference scores for each of the four tasks and the composite based on the three that formed a latent variable. The black “not” symbols indicate regression coefficients from the stepwise regression that have bootstrapped 95% confidence that include zero (*see* text for details). A blue border signifies a significant predicter of the incongruent trial RTs after congruent trial RTs have been regressed out. A lasso signifies a predictor that was significant in the LASSO regressions

It is also instructive to look at separate stepwise analyses for each of the four tasks. The outcome of the composite analysis and the outcomes for the four separate analyses are shown in Fig. 3. The names of the predictors included in the final stepwise model are shown in colored rectangles (together with their standardized regression coefficients). As shown in Fig. 3, Raven’s was the only predictor to enter the final model for all four tasks, and Sex was included in the final model for both the spatial Stroop and vertical Stroop tasks. Years of music training, team sports ability, and frequency of mindfulness/meditation were each represented in the model for a single task.

Assessing if the “significant” predictors with the smallest regression coefficients in the analyses of the individual tasks would be likely to replicate or if they emerged only because the stepwise regressions overfit the data is difficult. Three steps were taken to assess the reliability of the predictors shown in Fig. 3. First, for all of the stepwise regressions reported above, the final model was rerun as forced entry so that bootstrapped (1000 samples) 95% CIs could be derived for each of the regression coefficients using SPSS. As shown in Fig. 3 with the black “no” symbol, this analysis identified two regression coefficients with 95% CIs that included zero.

Another way of validating the stepwise regressions on the interference scores was to try to isolate the interference control that occurs on incongruent trials by treating the incongruent trial RTs as an outcome variable and to control for the processes shared by both trial types by entering congruent-trial RT in the first block and then stepping in the 11 predictors in a second block. This method removes the linear effects of the congruent condition, and the predictors of interest are regressed on the residuals (Cronbach & Furby, 1970; Pettigrew & Martin, 2014; Salthouse, 2010). The residuals indicate whether an individual’s performance on the incongruent condition is larger or smaller than would be predicted from their baseline score. Significant predictors in the regression analyses using incongruent trial RTs as the outcome variable are indicated in Fig. 3 by blue borders surrounding an embedded rectangle. For the composite measure, the same two predictors are identified: Raven’s (*β* = −.098, *p* < .001) and Sex (*β* = −.062, *p* = .005). Thus, two different methods for isolating interference control processes converge on the same regression model for the composite measure. However, as shown in Fig. 3, some inconsistencies exist between regressions on the interference scores versus the incongruent-trial RT residuals for the Simon task when considered separately; namely, the ability-at-team-sports predictor is significant only in the analysis of difference scores, and the mindfulness/meditation predictor is significant only in the analysis of the residuals.

A third approach was to rerun the analyses using LASSO (least absolute shrinkage and selection operator) regression (Tibshirani, 2011). The goal of LASSO is to obtain a subset of predictors that minimize prediction error by imposing a constraint on the model parameters that cause regression coefficients for some variables to shrink toward zero. The model with the lowest “overfitting” score is usually the best choice for predictive power. Yarkoni and Westfall (2017) advocate that testing predictive accuracy in a LASSO is a way to avoid complex models that potentially overfit noise, avoid inconsistencies in outcomes across studies, and avoid the need for complex theories to explain the complex pattern of results.

The set of significant LASSO predictors are tagged in Fig. 3 with a lasso. General agreement exists between the stepwise analyses of interference scores, incongruent-trial RT residuals, and the LASSO regression. But, in three cases, the LASSO models were simpler and “eliminated” a predictor: team sports and meditation in the Simon task and sex in the vertical Stroop task. However, a contrast in the opposite direction was present; namely, team sports (*β* = − 0.161) was a significant predictor in the LASSO regression of the composite interference scores but failed to enter the first two regressions.

### Bayes factors in our regression analyses

While interpreting the outcome of our regression analyses using the 11 predictors shown in Table 4 and the composite interference scores as the outcome variable we have tried to avoid the inference that the absence of a relation between interference control and a factor like music training is evidence that the effect is absent. The preponderance of null results merits the conclusion that there is no compelling evidence that a domain-general inhibitory control mechanism is enhanced by music training, meditation/mindfulness, bilingualism, video gaming, or exercise. To gain some sense of how the data provide evidence for the null versus alternative hypothesis we have used SPSS Bayesian Statistics for Linear Regression to explore Bayes Factor analyses on the composite interference scores. When all 11 predictors are included the R^2^ = .224 and yielded a BF of 7.86 in favor of the alternative. This is typically viewed as “substantial” evidence for the alternative. This BF is, of course, driven by Raven’s and sex. More interesting, when we tested a model that did not include Raven’s or sex (namely, a model consisting of bilingualism, music training, meditation, video gaming, exercise, team sports, age, immigrant status, and SES) the BF favoring the alternative was .001, a magnitude that is obviously inconclusive. A regression model that includes just the five activities (music, meditation, bilingualism, video gaming, and physical exercise) that have been hypothesized to enhance inhibitory control yielded an R^2^ of .065 with a BF = .007. This too is inconclusive. At the level of simple zero-order correlations the Pearson correlations between each of the five activities and the composite interference scores provide substantial evidence for the null in four cases: music training (BF = 13.5), mindfulness/meditation (BF = 15.1), bilingualism (BF = 9.8), and exercise (BF = 7.7). In contrast, the BF (0.3) for video gaming is inconclusive. However, recall that frequency of video gaming is higher for males and that video gaming is never included in a stepwise regression model that includes sex as a factor. In summary, the BF analysis supports that the Raven’s and sex are reliable predictors of composite interference scores, but it does not provide substantial evidence that the other predictors, considered as a set, are null. However, when treated as separate zero-order correlations most of the remaining predictors of the composite interference score have substantial evidence favoring the null.

### Further analyses of sex, gF, and congruency

To further explore the effect of sex on the composite interference scores a three-way (Sex, Task, & Congruency) mixed ANOVA was conducted and the Sex x Congruency interaction was significant, *F*(1, 199) = 10.59, *p* = .001, partial *η*^*2*^ = .051, indicating that interferences scores are larger for females than males. However, when both Raven’s scores and team-sports ability are included as covariates the Sex x Congruency interaction is no longer significant, *F*(1, 190) = 0.03, *p* = .863, partial *η*^*2*^ = .001. The disappearing interaction is difficult to interpret. Both Raven’s and team-sports ability significantly differ across sex with males having greater Raven’s scores (9.2 versus 8.2) and team-sports ability (3.2 versus 2.5). Thus, Miller and Chapman (2001) would argue that it is inappropriate to use Raven’s and team-sports ability as covariates and that the adjusted means are not trustworthy.

Another strategy for pulling apart the effects of sex and Raven’s on interference control is to match the males and females on Raven’s score. To that end we matched each of the 52 males to a randomly selected female with the identical Raven’s score. With one exception the matched pairs received the four tasks in exactly the same order. If the factor primarily responsible for producing a male advantage in the Raven’s test is the same factor producing a male advantage in the interference tasks, then the advantage should be attenuated or even eliminated in an analysis of the matched groups. However, in a mixed 2 × 2 × 2 ANOVA on the RT scores with sex as the grouping variable and congruency and task as repeated measures the Sex x Congruency interaction remained significant, *F*(1, 102) = 8.42, *p* = .005, partial *η*^*2*^ squared = .005.^7^ The interference effect for males in the full set of 201 participants was 25.3 ms smaller than that for females. In comparison the analysis of the 52 matched pairs showed a male advantage of 24.7 ms. It appears that the processes driving the male advantage in our interference scores are different from those driving the male advantage in Raven’s scores as the differences in interference control are equivalent in both the full and matched sample.

### Relationships between self-control, impulsivity, and interference effects

The three facets of impulsivity (premeditation, urgency, and perseverance) identified by Whiteside and Lynam (2001) that were included in the present study should moderately correlate with one another as factors nested under the higher order trait of impulsivity but only moderately as they have been shown to reflect different facets that enjoy some degree of separability. Table 5 shows that two of the three correlations are highly significant but that the correlation between urgency and perseverance was not, *r*(201) = −.081, *p* = .25. The Tangney, Baumeister, and Boone (2004) brief self-control scale (BSCS) was also expected to correlate with the three subscales of impulsivity. This was true in our sample with the strongest correlation between the facet of urgency and self-control: *r*(201) = −.608, *p* < .0001.

**Table 5:** Bivariate correlations between the impulsivity subscales of premeditation, urgency, perseverance, and the Tangney et al. BSCS

	Premeditation	Urgency	Perseverance
BSCS	+.326^b^	−.608^b^	+.385^b^
Premeditation		−.210^a^	+.491^b^
Urgency			−.081

^a^Correlation is significant at the .05 level (two-tailed)

^b^Correlation is significant at the .01 level (two-tailed)

The primary purpose of including these self-rating scales was to determine the degree to which they predict performance in the four nonverbal interference tasks. Furthermore, the interference score (rather than global RT or accuracy) was thought most likely to tap into the types of self-control captured in the trait measures. Table 6 shows the correlations between the four self-control scales, the interference effect in each of the four tasks, and finally correlations with the composite interference effect formed from the Simon, spatial Stroop, and vertical Stroop tasks. Perhaps the most important message delivered from Table 6 is how weak the correlations are between the measures of self-control/impulsivity and the interference effects that presumably reflect some type of conflict-resolution processing. Not one of these correlations was significant at *p* < .05.

**Table 6:** Correlations between the four self-control/impulsivity scales and the individual task and composite interference effects based on RT

Interference Effect	Premeditation	Urgency	Perseverance	BSCS
Simon	−.044	+.024	−.121	−.018
Spatial Stroop	−.062	+.047	−.119	−.016
Vertical Stroop	−.075	+.133	−.037	−.036
Flanker	+.014	+.100	+.015	−.062
Composite of 3	−.059	+.134	−.067	−.054

**Fig. 4:**
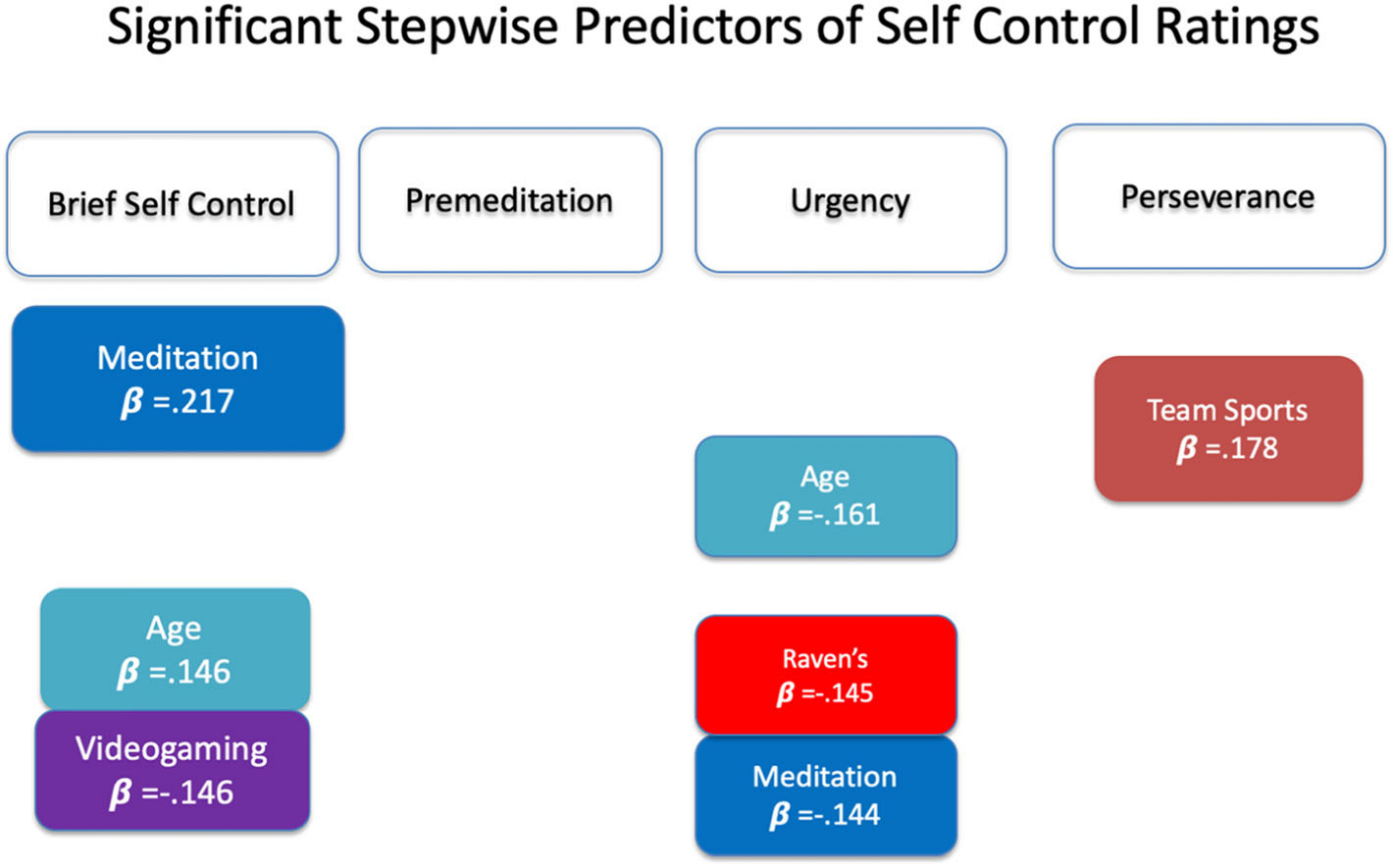
Significant predictors with their beta coefficients for the stepwise regression analyses on the self-control scores for each of the four scales

Given that the set of 11 variables used as predictors of the interference scores have all been hypothesized to be associated with inhibitory control, testing their ability to predict the self-ratings of cognitive control make sense. These are shown in Fig. 4. The variables (i.e., Raven’s and Sex) that are the most consistent in accounting for small but significant variance in interference scores derived from nonverbal tasks (*see* Fig. 3) play little apparent role in predicting the degree of self-reported cognitive control (Fig. 4).

## Discussion

### Relationship between self-control/impulsivity and interference control

Participants completed Whiteside and Lynam’s (2001) subscales for three facets of impulsivity (premeditation, urgency, and perseverance) and Tangney et al.’s (2011) BSCS. Earlier reviews and analyses by Allom et al. (2016) and Duckworth and Kern (2011) reported very small correlations between self-report trait measures of self-control and objective measures of EF obtained with a variety of laboratory tasks but did not specifically examine the nonverbal interference tasks that are the focus of the present study.

As described in more detail in the results, and as shown in Table 6, the correlations between the trait measures of impulsivity/self-control and the interference effects that presumably reflect some type of conflict resolution processing are nonsignificant. The strong and significant correlation reported by Enticott et al. (2006) between trait impulsivity and spatial Stroop interference was not significant in our data for premeditation, urgency, or perseverance (*see* Table 6). With the exception of Enticott et al., the cumulative evidence shows that interference effects do not predict self-reported impulsivity in everyday life. As Wolff et al. (2016) note, a persisting gap between EFs and self-control implies that adequate EF could be a necessary condition, but it is clearly not a sufficient condition for successful self-control.

Another potential cause of the disconnect may be that the laboratory tasks are very sensitive to the participant’s calibration of speed and accuracy, a skill that has little relevance to delaying gratification (urgency), planning before acting (premeditation), or having the grit to persist in the face of adversity (perseverance). Either implicitly or explicitly, the computerized EF tasks almost always encourage the participant to go as fast as possible without making more than an occasional error. The mechanisms needed to filter out competing information in the nick of time and when there is little intrinsic value associated with a “correct” response, may be different from those needed to resist actions that are affect laden and/or creatures of habit and have genuine costs and benefits. Moreover, competing information in the real world does not typically appear at random and is exquisitely tied to the onset of new task relevant information, and the conflict need not be resolved within the first couple of hundred ms of the onset of the event. In fact, any rapid suppression of responses counter to long-term goals often needs to be sustained in order to be ultimately successful.

### Relationship between special experiences and interference control

#### Bilingualism

As shown in Table 4, the correlation between the ratio of L2/L1 proficiency and the composite measure of interference control was near zero. For this dataset, Paap et al. (2019) also reported no significant relationships between interference control and any of the following dimensions of bilingual experience: L2 proficiency, similarity of L2 to L1, age-of-acquisition of L2, percentage of time speaking L2, frequency of language switching per day, frequency of code switching, the mean number of languages used per context (e.g., at home, at work, at school, with friends, etc.), and the number of languages spoken. The results from this study are consistent with the meta-analyses described earlier (Donnelly et al., 2019; Lehtonen et al., 2018; Paap, 2019). The most straightforward conclusion is that bilingualism does not enhance inhibitory control. Paap, Johnson, and Sawi (2015, 2016) present an extended discussion of why a steady drip of significant findings occurs in the published literature, and Paap et al. (2019) conclude that bilingual language control may be encapsulated within the language-processing system and, consequently, have no beneficial effect on domain-general control.

#### Video game playing

In the present study, the composite interference score significantly correlated with the frequency of video game play (r = − .214), but when Raven’s scores, sex, and other factors were entered into the model, the regression coefficient for video game playing was no longer significant. Likewise, the frequency of video game play was not a predictor in the regression analyses of the individual tasks. The regression results are consistent with the results of Dye et al. (2009), showing no difference between players and nonplayers on flanker effects and the results of Unsworth et al. (2015) analyses showing no correlation between a continuous measure of video gaming and either Simon effects or flanker effects. From the studies reviewed in the introduction, only the training study by Hutchinson et al. is consistent with the hypothesis that video game play improves interference control and that study was restricted to Simon effects. However, as shown in Fig. 3, frequency of video game play was not a significant predictor for Simon effects either. In summary, little exists in the present study to stem what appears to be the tide that video game play has little or no impact on interference control as expressed in nonverbal interference tasks.

#### Music training

Years of music training was not a significant predictor of the composite interference scores. Neither was it a significant predictor in any of the separate stepwise analyses of interference scores. However, it was a significant predictor of Simon incongruent-trial residuals. This was the first time that the relationship between music training and Simon effects was assessed, and accordingly, no prior literature exists to support or guide an interpretation that music performance may hone interference control in the Simon task but not produce benefits on other nonverbal interference tasks. Consistent with the expectations laid out in the introduction, the current results provide no compelling evidence that music training or performance enhances inhibitory control to the extent that this hypothesis can be confirmed across a set of nonverbal interference tasks.

#### Mindfulness /meditation

meditation/meditation in our data are very inconsistent. The bivariate correlation between frequency of meditation and the composite interference scores was near zero (r = + 0.05), as was the beta coefficient for the regression analysis on the composite interference scores (β = + 0.07). However, significant positive beta coefficients were found for the meditation/mindfulness predictor in both the stepwise analysis of spatial Stroop interference scores (β = + 0.14) and the stepwise analysis of spatial Stroop residuals (β = + 0.07). These positive regression coefficients are, of course, opposite of what one would predict if mindfulness/meditation led to smaller interference scores and faster incongruent trials. The reliability of these positive regression coefficients in the analysis of the spatial Stroop is further questioned by the finding that the bootstrapped 95% CIs for both regression coefficients included zero. In contrast, in the analysis of the incongruent RT residuals for the Simon task, the beta for the mindfulness/meditation predictor was significant and in the expected negative direction (β = − 0.06). However, it was not a significant predictor of either the stepwise or LASSO regressions on Simon interference scores, which reduces the impact of the positive outcome in the regression on the Simon incongruent-RT residuals.

Recall that many training studies did not show significant facilitation and that most of the cross-sectional comparisons of meditators to non-meditators showed no group differences. We offer the following conjecture regarding why this pattern occurs in studies of mindfulness /meditation. Potential effects of bilingualism, music performance, or playing video games on nonverbal interference tasks are clear cases of far transfer in the sense that, for example, musicians are not practicing music when they are doing a flanker task, but meditators may be in a meditative state. This seems more probable when the last session of training culminates with the post-test of the interference task. Whether intentional or not, if a meditative state continues into the post-test, all types of cognitive control may be enhanced. Posner (2018) has recently reported that connectivity in the anterior cingulate cortex is improved following 2 to 4 weeks of meditation training and that the increase in frontal theta following meditation training might be the cause of improved connectivity. A critical question is whether improved connectivity is relatively durative and facilitates any processing employing those networks or if meditation induces temporary states that must be reinstated to produce benefit.

#### Team-sports ability

Team-sports ability was self-rated using this item originally developed by Paap and Greenberg (2013): *Team sports often involve dividing your attention between a ball, a goal, your opponents, and your teammates. Do you excel at these sports?* Team-sports ability enjoys the third highest zero-order correlation (r = − 0.19), with the composite interference scores and the beta coefficient being significant in the analysis of Simon interference effects (β = − 0.19). However, it did not enter the final stepwise model for any of the other tasks or for any of the tasks in the regression analyses of incongruent RT residuals.

In regression analyses similar to those used in the present study, Paap and Greenberg reported significant beta coefficients in their Study 3 for separate analyses of flanker effects and switching costs but not for Simon effects. A further complication to the interpretation of the relationship between sport’s ability and inhibitory control is that males rated their sports ability higher than females, and as reported above, these nonverbal interference tasks often produce male advantages.

A possible relationship between team-sports ability and interference control may be surprising for those familiar with contemporary theories in sports psychology because of the emphasis on the role of deliberate practice leading to automatization of skilled sport performance (e.g., Ericsson, Charness, Feltovich, & Hoffman, 2006). However, Toner and Moran (2014) have advocated for more research on the role of controlled processing and Furley and Wood (2016) review evidence that working memory capacity is often associated with better performance in team sports. The study most related to the type of interference control that is the focus of the present investigation is that of Vestberg, Gustafson, Maurex, Ingvar, and Petrovic (2012), who tested soccer players with different levels of advanced skills using the D-KEFS test battery of executive functions (Homacka, Lee, & Ricco, 2005). The design fluency component requires participants to remember previous responses by updating working memory and inhibition skills in order to not repeat previous responses. Also included was a color-word Stroop test and the Trail-Making Test. Players from the Swedish highest national soccer leagues outperformed players from the lower division on all of these measures of EF. Furthermore, the EF test scores obtained in the fall of 2007 were used predict a performance measure that combines goals and assists over a 17-month interval in 2008 and 2009. The correlation (*cf* = 0.54, *p* = .006) was statistically significant and noteworthy in magnitude. These results are consistent with the interpretation that EF contributes to team-sports ability, even at very high levels of skill.

#### Physical exercise

Individuals with superior team-sports ability are also likely to be fit, and in the present study, the frequency of exercise, working out, and participation in team sports notably did not predict the composite interference scores or the outcome measure in any of the task-specific regression analyses. Furthermore, these small correlations are positive, rather than negative, indicating that individuals reporting higher levels of physical exercise were actually trending toward larger interference effects.

#### SES

In several large-scale studies (Paap et al., 2017; Paap & Greenberg, 2013; Paap & Sawi, 2014), the correlations between parents’ educational levels and a variety of EF measures were always nonsignificant and often near zero. The participants in each case were university students. In the present study, the proxies for SES were extended to include family income. Neither the composite measure of SES nor the separate factors predicted the composite interference scores. Studies using children often report effects of SES on EF. For example, Calvo and Bialystok (2014) tested six-year-old children and reported main effects for both bilingualism and SES on the flanker and Stroop effects. A possible explanation for why the relationship is consistently weak and nonsignificant in our studies is that the lower SES students in our college student population either had enriching early experience despite their parent’s education and income or have otherwise managed to compensate for disadvantages in early childhood.

### The conundrum of sex, sports, gF, and their relationship to interference control

Males had smaller interference scores in the composite measure and individual regression analyses of the spatial and vertical Stroop task. Although sex was confounded with Raven’s scores, the same male advantage was observed when the 52 males were matched in Raven’s to 52 females. This evidence for sex differences in interference control in the present study should be interpreted cautiously, but two recent studies using spatial Stroop tasks similar to ours also reported statistically significant male advantages in the form of smaller interference effects. Stoet (2016) tested 236 males and 182 women in an online study and reported 42 ms interference scores for males and 29 ms for females. Evans and Hampson (2015) tested 90 males and 86 females and, estimating from their Fig. 4, the interference effects were apparently approximately 60 ms and 40 ms, respectively. For purposes of comparing across the studies, a separate two-way ANOVA on our spatial Stroop RT data yielded a significant Sex x Congruency interaction (*F*(1, 199) = 14.92, *p* < .001, partial *η*^*2*^ squared = .070). The interference effect for males was 70 ms compared to 96 ms for females. The overall spatial Stroop effects in our study are atypically large. This is not too surprising as only 25% of the trials were incongruent compared to the usual 50–50 balance. A more extreme bias was used by Christakou et al. (2009) with only 11.5% incongruent trials and led to even larger spatial Stroop effects, namely, 110 ms for males and 129 ms for females. This male advantage was not statistically significant,^8^ but the study was underpowered with only 38 males and 25 females. When incongruent trials are rare, a strategy of relying entirely on reactive mechanisms may be induced. Further pursuit of the sex effect in the spatial Stroop task with a systematic manipulation of the proportion of incongruent trials and determination of whether the male advantage is nested primarily in a preference for reactive inhibition over proactive may be worthwhile.

Lynn and Irwing (2004) suggest that the male advantage in the Raven’s test may be nested in the spatial-visualization ability in hierarchical factor models like Carroll’s (1993). In contrast to Raven’s, the ability to manipulate visual-spatial representations may play little role in interference tasks that require decisions about a single stimulus (e.g., spatial Stroop, vertical Stroop, and Simon) that remains in view until a response is made Although quite speculative, this provides one explanation for why matching on Raven’s scores does not reduce or eliminate the male advantage in interference control.

The Raven’s test was developed to assess an individual’s abstract reasoning ability without having to rely on declarative knowledge and the influence of language, education, or cultural factors (Carpenter, Just, & Shell, 1990; Raven, 1939). As reviewed by Lynn and Irwing (2004), many experts judge it as one of the best tests of gF as defined by Cattell (1971) because of its ability to discriminate relations, reason abstractly, solve novel problems, and adapt to new situations. Paap and Sawi (2014) note that EF should be related to gF because the components of EF (monitoring, updating, switching, and inhibiting) logically serve successful reasoning, problem solving, and adapting, whereas high quality reasoning seems to require more than the sum of the parts of EF. However, the degree to which EF and gF are actually separate constructs has been questioned, if not challenged, by Salthouse (Salthouse, Atkinson, & Berish, 2003; Salthouse, Pink, & Tucker-Drob, 2008) who showed that multiple measures of gF were strongly related to several measures of EF and that performance on classic EF tasks will sometimes load on the gF factor rather than the EF factor when allowed to do so. Salthouse (2010) observes, in a somewhat dispiriting manner, that if gF encompasses a broad spectrum of controlled processing, then investigators working from different research traditions may be giving different names to the same dimension of individual differences. That said, the intimate relationship between EF and gF appears less promiscuous for the inhibiting function of EF than for updating (Salthouse et al., 2003, Tables 9 and 10). This would be consistent with a working hypothesis that the interference effects measured in the present study and Raven’s scores share some dimensions of individual differences, but are separable constructs.

Recall that in the present study males outperformed females on the Raven’s test. Setting aside the omnipresent possibility of a Type 1 error, the difference could be due to a bias favoring higher gF males in our student population or it could reflect a genuine difference in the general population of young adults. Although the presence of sex differences in the Raven’s test remains controversial, Lynn and Irwing’s (2004) meta-analysis of 57 studies showed a statistically significant male advantage emerging at the age of 15 (0.10d) that grew to 0.33d among young adults aged 20–29 and remaining stable through old age. Their meta-analysis had two notable strengths: (1) avoiding apples and oranges comparisons by including only versions of the Raven’s test and excluding other intelligence tests and (2) including only general population studies with samples of at least 50 males and 50 females.

### Limitations

Although four different nonverbal interference tasks were used that varied in terms of S-S compatibility and whether conflict arose from distractors versus a task-irrelevant dimension of the imperative stimulus, some results possibly would be different if the proportion of incongruent trials encouraged greater reliance on proactive inhibition. Likewise, some of our background variables relied on a single item. Future research might focus on developing scales for these predictors that have desirable psychometric properties. The complete absence of significant relationships between interference scores and measures of self-control and impulsivity may be attributed, in part, to the reliance on self-reports that rely on memory and are subject to various types of bias.

### An optimist’s conclusions

The interference scores from the four nonverbal interference tasks have adequate split-half reliabilities and three (i.e., Simon, spatial Stroop, and vertical Stroop) cohered into a latent variable that may reflect the ability to resolve conflict between two dimensions of a single stimulus (namely, identity and location). This latent variable, expressed as a standardized composite of each task’s interference scores, is significantly related to sex and gF in that males and individuals with higher intelligence are better at resolving this type of conflict. The male advantage is sustained in a subset of males and females that are matched on Raven’s scores. Years of musical experience did not predict the composite interference scores but was associated with the magnitude of the Simon effect in incongruent RT residuals. As the Simon task is a pure S-R task (*see* Fig. 1), it may be more sensitive to a form of conflict resolution common to music performance, although we have no reason to believe that music performance is richer in S-R incompatibilities compared to S-S. Future research could test this hypothesis. Likewise, frequency of mindfulness/meditation did not predict the composite interference scores, but its regression coefficient was significant in predicting both Simon and spatial-Stroop effects. In the previous research (*see* Table 1), the relationship between mindfulness/meditation and interference control appears more consistent in the training studies than in studies comparing meditators to non-meditators. Thus, the possibility that mindfulness/meditation enhances interference control remains a plausible hypothesis but may be more robust following training. Finally, a surprising disconnect exists between the composite measure of interference control and self-ratings of impulsivity and control in everyday life.

### A pessimist’s conclusions

The problem with the conclusions offered by optimists is that they are often influenced by a confirmation bias for reporting positive effects and a penchant for seeing any positive findings as a roadmap to future research that might eventually validate the constructs of interest, albeit with a more complicated theory than initially envisioned. But if the constructs do not exist or are markedly different, then the roadmap is a blind alley that prevents self-correction. Therefore, a pessimist might offer a different conclusion.

Four common nonverbal interference tasks that are typically assumed to measure inhibitory control did not all load on a common latent variable. The three tasks that did form a latent variable were not the tasks one would expect on the basis of Kornblum’s taxonomy (*see* Paap et al., 2019). Prior to the present study, no latent variable analysis has been able to extract a latent variable that includes the interference scores from two or more nonverbal interference tasks.^9^ When prior studies do succeed in extracting a latent variable that includes a single nonverbal interference score, it loads weakly and is dominated by a different measure—often the antisaccade task (Rey-Mermet et al., 2018). In the same vein, Friedman and Miyake (2016) could not extract an inhibition factor that was separable from updating and shifting.

The formation of a latent variable for three of our tasks could be an artifact of the stimulus and response similarities across the tasks. Rey-Mermet et al. (2018) recommended and practiced the advice to deliberately introduce differences in the stimulus displays and response modes for tasks selected to load on the same latent variable. As Friedman and Miyake (2016) noted, task impurity seems to be an unavoidable quality of EF tasks like the nonverbal interference tasks. By definition, EFs involve controlling lower-level processes, so any inhibitory control task must include nonexecutive processes that could influence performance in addition to the EF of interest. One method for removing the influence of unreliability and task impurity is latent variable analysis. For present purposes, the important characteristic is that they capture only common variance across multiple measures; this common variance cannot include random measurement error and will not include non-EF variance to the extent that tasks are selected to have different lower-level processes. The perceptual encoding, response selection, and response execution processes in the present study are, unfortunately, very similar and very well could explain the significant but small intertask correlations.

With the regression analyses, when a set of 11 predictors that have been hypothesized to be related to inhibitory control were entered in a stepwise regression on the composite interference scores, only sex and Raven’s score entered the model. When the same stepwise regression was conducted on the interference-scores from each individual task, Raven’s score was the only significant predictor for all four tasks. Sex was included in the model for two of the tasks with music training, mindfulness/meditation, and team sports included in only one model. Two of these predictors in the bootstrapped analysis of individual tasks had 95% CIs that included zero and are likely to be unreliable in future tests. The three methods (stepwise regression on interference scores, hierarchical regression on incongruent trial RT, and LASSO) intended to provide converging evidence each identify a predictor that the other two do not: music is selected in the analysis of incongruent-trial RT residuals (Simon task), team sports is selected by the stepwise regression of the interference scores (Simon task), and team sports is selected by the LASSO regression (composite of 3 tasks). The only solid relationship is that Simon, spatial Stroop, and vertical Stroop effects decrease as the Raven’s scores increase. Taking at face value that Raven’s is tapping into gF abilities and not skills, this would suggest that interference control in these generic nonverbal tasks are, at the individual differences level, influenced more by heritability than experience (*see* Paap, 2018b for a discussion of the possible role of heritability in EF).

The possibility of a causal relationship between EF and gF is important, as illustrated by the Engle, Kane and colleagues theory that EF/EA drives both gF and WMC. But the only nonverbal interference task typically included in their EA battery is the flanker task, and the flanker effect always loaded weakly on the EF/EA latent variable. A related but different issue was raised by Chuderski et al. (2012), who reported that latent variables for both inhibition and interference did not account for any meaningful portion of gF variance because the simple correlations were completely mediated by the storage capacity latent variable. The *coup de grâce* that inhibitory control is related to gF may be the Rey-Mermet et al. (2019) finding that a coherent latent variable for EF could not be established despite good reliabilities for all measures. Furthermore, WMC and gF—modeled as separate but correlated factors—were unrelated to the individual measures of EF, which included modified versions of both the arrow flanker and Simon tasks. In summary, inhibitory control is probably task-specific, not domain-general, and not causally related to gF. At best, subsets of nonverbal interference tasks may exist that share more specific mechanisms of conflict resolution. Going forward, we should stop using the flanker, Simon, and spatial Stroop tasks.

Another major purpose was to further evaluate the relationship between trait measures of self-control or impulsivity and measures of inhibitory control that are commonly used in cognitive psychology laboratories. Although the array of nonverbal interference tasks used in the present study was different from most of the cognitive control tasks surveyed by Duckworth and Kern (2011), our results sustain their conclusion that trait-like measures of self-control and interference control measured in RT tasks are not measuring the same thing. The differences in temporal dynamics and motivation may contribute to this dissociation. In any event, one should not interpret interference scores as “inhibitory control,” “self-control,” or “impulsivity” without converging evidence supporting such a generalization.

## Data Availability

The datasets generated and/or analyzed during the current study are available from the corresponding author on reasonable request.

## Appendix

**Table 7:** Distributional characteristics of the predictor and outcome variables across 201 participants

Variable	Min	Max	Mean (SD)	Skew (SE)	Kurtosis (SE)
Raven’s scores	2	12	8.4 (2.3)	−.56 (.17)	−.21 (.34)
Teams sports ability	1	5	2.7 (1.1)	+.01 (.17)	−.75 (.34)
Frequency of physical exercise	1	5	3.2 (1.0)	−.11 (.17)	−.41 (.34)
Years of musical training	0	32	2.7 (4.3)	3.1 (.17)	13.9 (.34)
Frequency of playing an instrument	1	5	1.8 (1.0)	1.3 (.17)	1.37 (.34)
Frequency of playing video games	1	5	2.2 (1.1)	.78 (.17)	.03 (.34)
Frequency of meditation or mindfulness	1	5	2.4 (1.0)	+.43 (.17)	−.42 (.34)
L2/L1 ratio	0	1	.53 (.37)	−.34 (.17)	−1.38 (.34)
SES (parents’ education & family income)	1.3	7.3	4.4 (1.3)	−.05 (.17)	−.63 (.34)
(Lack of) Premeditation	1.8	4.0	3.1 (0.4)	−.18 (.17)	+.09 (.34)
Urgency	1.2	3.9	2.3 (0.5)	+.11 (.17)	+.17 (.34)
(Lack of) Perseverance	2.1	3.9	2.9 (0.3)	−.00 (.17)	−.08 (.34)
Self-Control (BSCS)	1.8	4.8	3.3 (0.6)	+.03 (.17)	−.17 (.34)
Z Composite RT across four tasks	−1.3	2.5	0 (.65)	.84 (.17)	1.03 (.34)

**Table 8:** Bivariate correlation matrix for set of 11 predictors and four outcome variables

Variable	2	3	4	5	6	7	8	9	10	11	12	13	14	15
1. Raven’s	+.19^b^	−.13	−.07	+.14^a^	+.22^b^	+.08	−.12	−.10	+.07	−.16^a^	−.26^b^	−.27^b^	−.31^b^	−.11
2. Sex		+.10	−.13	+.17^a^	+.47	+.14^a^	+.15^a^	+.11	+.29^a^	−.17^a^	−.15^a^	−.26^b^	−.21^b^	−.05
3. Age			+.06	−.10	+.01	+.07	+.01	+.04	−.14	−.15^a^	+.14^a^	+.07	+.02	−.00
4. Immigrant				+.02	−.05	+.02	−.08	−.01	−.11	+.39^b^	+.08	+.15^a^	+.12	−.05
5. SES					+.12	+.11	+.11	+.03	+.12	−.23^b^	−.14^a^	−.08	−.07	+.00
6. Video games						+.00	+.09	+.11	+.20^a^	−.16^a^	−.14	−.14^a^	−.18^b^	−.09
7. Music							+.15^a^	+.05	+.02	−.16^a^	−.26^b^	−.06	−.09	+.05
8. Exercise								+.38^b^	+.32	−.08	−.04	+.01	+.10	+.08
9. Meditation									+.28^a^	−.01	−.18^a^	+.11	−.01	−.02
10. Team sports										−.08	−.21^b^	−.13	−.14^a^	−.16^a^
11. L2/L1 ratio											+.11	+.09	+.11	−.04
12. Simon												+.36^b^	+.26^b^	+.08
13. Spatial Stroop													+.42^b^	+.10
14. Vertical Stroop														+.17^a^
15. Flanker														

^a^Significant at the *p* < .05 level

^b^Significant at the *p* < .01 level

## Footnotes

1 The ANT is a basic flanker task but with the addition of temporal and spatial cues that enable the calculation of individual difference measures for “alerting” and “orienting” as well as of the interference score, which is often referred to as executive attention.

2 In the number Stroop, participants are asked to report the number of digits in a row while ignoring the identity of the digits.

3 In the Go/no-go task, participants were asked to press a button as soon as possible when a stimulus appears (go trials), except when an “X” is presented, in which case they should withhold a response (no-go trials).

4 In the antisaccade task, participants are asked to identify a stimulus which is presented very briefly on the side opposite of a flashing cue. Thus, to perform this task successfully, participants have to suppress the reflexive saccade to the cue and perform a saccade in the opposite direction to identify the stimulus.

5 In tower-of-London (sometimes called tower-of-Hanoi) participants are given a starting configuration of rings on a set of pegs and instructed to make the starting configuration look like a target configuration by always moving one ring at a time and never placing a larger ring on a smaller ring.

6 In the trail-making test participants must connect, alternatively, numbers and letters in ascending order, and the measure divides the total time in the alternating condition to the total time required to connect numbers only.

7 The results were very similar when efficiency scores rather than RTs were used. Thus, the Sex x Congruency interaction in mean RT does not appear to be due to males and females adopting different strategies for titrating speed and accuracy.

8 In contrast to the behavioral results, significant group differences existed in the fMRI BOLD measures: females relied more on functional frontal mechanisms, whereas males relied more on functional parietal mechanism.

9 Kane et al. (2016) successfully extracted a latent variable for “attention constraint,” but all the tasks were versions of the flanker task. Furthermore, the authors characterize these tasks as performing “poorly overall,” as the measures correlated weakly across tasks.
